# Breaking myths and building awareness: spinal anesthesia for cesarean section in Palestine—what women know and what holds them backs- a cross- sectional study

**DOI:** 10.1186/s12871-025-03342-1

**Published:** 2025-09-30

**Authors:** Saleh Juneidi, Islam Jadallah, Mohammed Saleh, Bayyena Abu-Radwan, Haya Abu Mayyaleh, Ahmad Fasfoos, Dirar Zatari

**Affiliations:** 1 Faculty of Medicine, Hebron University, Hebron, Palestine; 2 Department of anesthesiology, Al-Ahli Hospital, Hebron, Palestine

**Keywords:** Spinal anesthesia, Cesarean section, Maternal awareness, Obstetric anesthesia, Patient knowledge, Anesthesia choice, Pain management

## Abstract

**Background:**

Cesarean section (CS) is one of the most frequently performed surgical procedures worldwide. Spinal anesthesia (SA) has become the preferred anesthesia technique for CS due to its advantages over general anesthesia, including better maternal hemodynamic stability and improved neonatal outcomes. However, maternal knowledge and attitudes toward SA significantly influence decision making and perioperative experiences. This study aimed to assess the knowledge, awareness, and attitudes of Palestinian women toward SA during CS.

**Methods:**

A cross-sectional study was conducted among Palestinian women aged 18–60 years who met the inclusion criteria and voluntarily provided informed consent. Data were collected using a structured, validated questionnaire distributed through social media platforms and maternity wards in Palestinian hospitals. A sample size of 384 participants was calculated using OpenEpi, with a 95% confidence level and a 5% margin of error. Statistical analysis was performed using SPSS version 26.

**Results:**

A total of 415 Palestinian females were included. The mean age was 27.3 ± 6.8 years. More than half expressed a preference for spinal anesthesia (SA) over general anesthesia (GA) for cesarean delivery. Knowledge levels were generally low, with less than 10% demonstrating high awareness. Although 63% had heard of regional anesthesia, fewer were familiar with SA specifically. Misconceptions were frequent; the majority failed to identify the correct injection site, and over half were unaware that SA could be used during active labor. While most participants recognized that SA allows maternal consciousness during surgery, less than one-third correctly identified its reduced fetal exposure compared to GA. Among those expressing concerns, the most commonly reported barriers were fear of chronic back pain, headaches, permanent paralysis, painful injection, intraoperative awareness, nausea or vomiting, and hypotension.

**Conclusion:**

Although a significant proportion of Palestinian women were willing to receive spinal anesthesia, many exhibited concerns and misconceptions about its safety and effects. These findings highlight the need for improved patient education and counseling on SA to enhance maternal confidence and decision-making. Addressing these concerns through targeted awareness programs could lead to better patient experiences and outcomes in cesarean deliveries.

## Introduction

Cesarean section (CS) is a surgical procedure that involves delivering one or more babies through an incision in the mother’s abdomen [1]. It stands as one of the most frequently performed surgeries globally according to the Organization for Economic Co-operation and Development (OECD) [2] and is also the most common obstetric operation emphasizing its pivotal role in modern obstetric and surgical practice [3]. A key component of a successful CS is the administration of anesthesia, which not only provides pain relief but also contributes to overall maternal well-being during and after the procedure [4]. 

Anesthesia plays a crucial role in ensuring a safe and pain-free experience during cesarean section (CS) procedures, Both general anesthesia and regional anesthesia are commonly used techniques for this purpose [5]. However, when considering the risks and benefits for both the mother and the fetus, regional anesthesia is generally the preferred approach [6]. Specifically, spinal anesthesia has gained increasing preference in recent years. It offers significant advantages, particularly in reducing maternal and risks, including better hemodynamic stability and fewer complications compared to general anesthesia [7]. Additionally, neonatal outcomes are improved, as spinal anesthesia avoids the systemic effects of general anesthesia, leading to safer conditions for the fetus [8]. Given these advantages, females knowledge of spinal anesthesia are essential, to have a less stressful experience during the procedure.

Maternal knowledge and awareness of spinal anesthesia are critical for informed decision-making during Cesarean section procedures. When women are well-informed about the benefits, risks, and expected outcomes of spinal anesthesia, they are more likely to make decisions that align with their preferences, which can enhance their overall experience [9]. However, without adequate knowledge, women may feel anxious or uncertain about the procedure, potentially leading to a less satisfactory experience or even the rejection of an otherwise safe method [10]. 

Women’s attitudes toward the type of anesthesia are often shaped by a complex interplay of personal beliefs, cultural factors, previous experiences with medical procedures, occupational status and preoperative information. Research has shown that fear of pain or perceived risks associated with regional anesthesia may lead women to express concerns or refuse this method. in addition, fear of regional block not working, needle phobia and Fear seeing things during surgery are additional causes [11, 12]. These misconceptions may contribute to delayed patient presentation, potentially negatively impacting outcomes and the quality of care [13]. 

Due to the importance of understanding the rule of spinal anesthesia during CS and its impact on patient choice and experience, the aim of this research is to assess the level of knowledge, awareness, and attitudes of Palestinian women toward spinal anesthesia during cesarean section. By assessing the current level of understanding and identifying misconceptions, the study seeks to provide insights that could enhance patient education and improve decision-making, ultimately leading to better experiences during cesarean deliveries in Palestine.

## Methodology

### Study design and population

This cross-sectional study targeted Palestinian females aged 18 to 60 years who met the inclusion criteria and voluntarily provided informed consent to participate. The population consisted of Palestinian women residing in Palestine who were eligible to participate in the study based on the defined criteria.

### Study time and setting

Data collection took place over a specified period, with questionnaires distributed through both social media platforms and in maternity wards across hospitals in Palestine. The study focused on assessing Palestinian women’s knowledge, awareness, and attitudes toward spinal anesthesia during cesarean section. A pretested and validated questionnaire was used [14], which was developed following an extensive literature review and consultations with experts including anesthesiologists, obstetricians, and psychologists. The questionnaire was divided into three sections: the first gathered socio-demographic information; the second evaluated the women’s knowledge about spinal anesthesia for cesarean deliveries, with 11 multiple-choice questions and a scoring system based on correct answers; and the third explored potential barriers to spinal anesthesia using a three-point Likert scale. A pilot study was conducted with 30 participants to test the reliability and validity of the questionnaire.

### Sampling and sample size

The sample size was determined using the online tool OpenEpi, with assumptions of a 50% prevalence of adequate knowledge about spinal anesthesia, a 95% confidence level, and a 5% margin of error. Based on these parameters, a minimum sample size of 384 participants was calculated.

#### Inclusion criteria

Female individuals aged 18 to 60 years who are Palestinian women residing in Palestine. Additionally, participants must have voluntarily provided informed consent to take part in the study.

#### Exclusion criteria

Females under 18 years of age or above 60 years of age, women with psychiatric disorders or cognitive impairments, women residing outside of Palestine or of non-Palestinian nationality, and women employed in the healthcare sector, including doctors and nurses.

### Variables

#### Dependent variables

The level of knowledge regarding spinal anesthesia for cesarean section, women’s concerns about spinal anesthesia, and their attitudes and decision-making related to spinal anesthesia. These factors will be assessed to understand how women perceive and make decisions regarding this method of anesthesia during childbirth.

#### Independent variables

Including age, educational level, occupation, marital status, number of children, and the number of cesarean sections (Cs) previously undergone. Additionally, monthly family income, the influence of healthcare providers, and concerns about potential adverse effects will be considered. These factors are expected to play a significant role in shaping participants’ knowledge, concerns, and attitudes towards spinal anesthesia for cesarean section, influencing their decision-making processes.

## Result

Table 1 presents the demographic characteristics of the participants. Most of them were in the younger age groups, particularly between 18–25 years. The majority were married and held at least a bachelor’s degree. A substantial portion of the participants had 1–3 children, while over one-third reported no children. Cesarean history was more common among a minority, and income levels were relatively balanced across three categories. When asked about their future anesthesia preference for cesarean sections, more than half preferred spinal anesthesia (SA) over general anesthesia (GA).

**Table 1:** Demographic characteristics of population (*N*= 415)

Variable	Category	*N* (%)
Age group (years)	18–25	205 (49.4)
	26–35	121 (29.2)
	35–45	59 (14.2)
	>45	30 (7.2)
Marital status	single	121 (29.2)
	Married	283 (68.2)
	Divorced or Window	11 (2.7)
Educational level	Uneducated	10 (2.4)
	School education	87 (21)
	Bachelor degree	298 (71.8)
	Post-graduate education	20 (4.8)
Number of children	none	151 (36.4)
	1–3	157 (37.8)
	>3	107 (25.8)
Number of cesareans	none	263 (63.4)
	1–2	107 (25.8)
	>2	45 (10.8)
Total monthly family income	<1500	64 (15.4)
	1500–3000	177 (42.7)
	>3000	174 (41.9)
Would you choose spinal anesthesia over GA if you have CS in the future?	Yes	243 (58.6)
	No	172 (41.4)
Hearing and source of Knowledge about Spinal anesthesia	No hearing about SA	82 (19.8)
	Specialist (Gynecologist/Anesthesiologist)	126 (30.4)
	Friend	102 (24.6)
	Internet	70 (16.9)
	Others	35 (8.4)

**Fig. 1:**
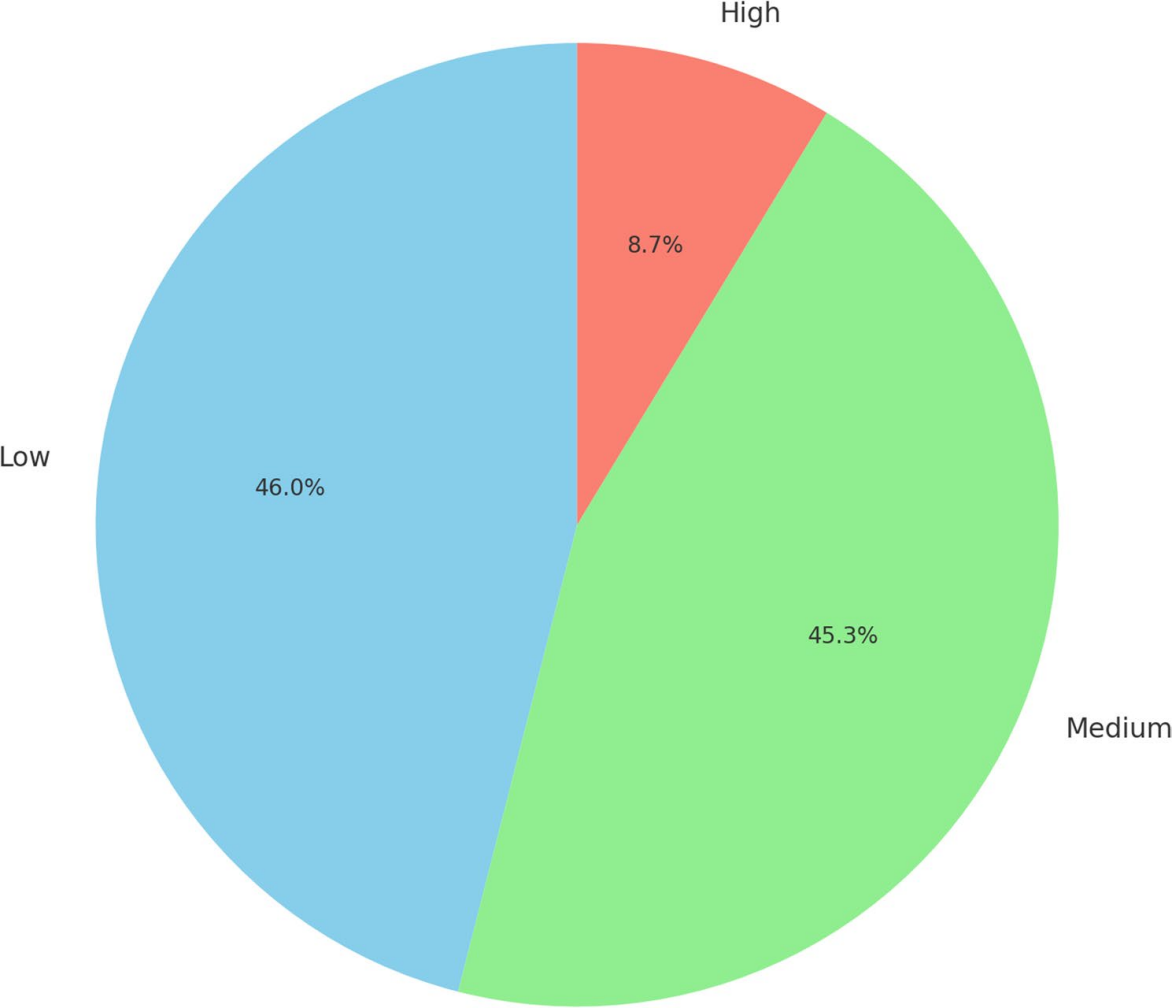
Level of knowledge of spinal anesthesia for cesarean delivery

As illustrated in Fig. 1, the level of knowledge regarding SA for cesarean delivery was mostly low to medium, with only a small proportion of participants demonstrating high knowledge.

Table 2 provides further insights into specific areas of knowledge and common misconceptions. A limited number of participants correctly identified the injection site as the lower back, though awareness was better regarding patient positioning during the procedure. There was considerable variation in responses regarding sensory perception and the transfer of anesthesia to the baby, with many participants lacking clarity in these areas. Most participants were aware that mothers remain conscious during SA, but uncertainty was evident regarding its safety in bleeding disorders and its possible administration during active labor. Although many participants were knowledgeable about the rapid onset of SA, misconceptions persisted about the duration of its effects and the role of nurses in administering it.

**Table 2:** Assessment of knowledge of spinal anesthesia for cesarean delivery among Population (*n* = 415)

Knowledge statement	*N* (%)
1. The injection site is at the following:	
Lower back*	104 (25.1)
Middle back	303 (73)
Upper back	8 (1.9)
2. Is the mother’s position during the injection lying on her side or sitting?	
Yes*	219 (52.8)
No	47 (11.3)
I don’t know	149 (35.9)
3. In spinal anesthesia, the patient can feel the sensations of touch and pressure.	
Yes	151 (36.4)
No*	140 (33.7)
I don’t know	124 (29.9)
4. In spinal anesthesia, the amount of anesthesia medication that reaches the baby is higher than in general anesthesia?	
Yes	18 (4.3)
No*	143 (34.5)
I don’t know	254 (61.2)
5. In spinal anesthesia, the mother stays conscious during the surgery?	
Yes*	361 (87)
No	13 (3.1)
I don’t know	41 (9.9)
6. Is spinal anesthesia a suitable option if the mother has any coagulopathy (bleeding disorder)?	
Yes	116 (28)
No*	60 (14.5)
I don’t know	239 (57.6)
7. Generally, general anesthesia is safer than spinal anesthesia?	
Yes	97(23.4)
No*	169 (40.7)
I don’t know	149 (35.9)
8. The effect of spinal anesthesia continues for a couple of days?	
Yes	87 (21)
No*	224 (54)
I don’t know	104 (25.1)
9. Spinal anesthesia can’t be given if the mother is in active labor?	
Yes	70 (16.9)
No*	118 (28.4)
I don’t know	227 (54.7)
10. The effect of spinal anesthesia will usually start after a couple of hours?	
Yes	47 (11.3)
No*	248 (59.8)
I don’t know	120 (28.9)
11. Nurses can give spinal anesthesia injections?	
Yes	32 (7.7)
No*	278 (67)
I don’t know	105 (25.3)
Total knowledge score (mean ± SD)	5.45 ± 2.41

* Indicates correct answer

**Fig. 2:**
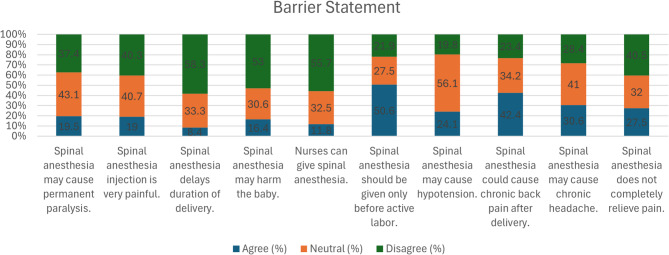
Assessment of barriers toward spinal anesthesia for cesarean delivery among population

Figure 2 explores participants’ beliefs and concerns related to SA. A substantial portion remained neutral or expressed disagreement regarding fears such as permanent paralysis or injection pain. Most participants did not associate SA with delayed delivery or harm to the baby. However, beliefs about when SA should be administered varied. Concerns related to side effects such as hypotension, chronic back pain, and headaches were present but often expressed with uncertainty. There was also a prevalent belief that SA effectively relieves pain, although not universally accepted.

Table 3 shows the association between socio-demographic variables and knowledge scores. Statistically significant differences were observed with age, marital status, number of children, cesarean history, monthly income, preference for anesthesia type, and source of information. Notably, individuals aged 35–45, married participants, and those with prior cesarean experience tended to have higher knowledge scores. Knowledge was also significantly higher among those who preferred SA and those who received information from medical professionals or reputable sources such as the internet, compared to those with no prior exposure.

**Table 3:** Differences in knowledge scores according to population socio-demographic characteristics

Variable	Knowledge score Mean ± SD	*P*-value
Age group (years)		0.001
18–25	5.09 ± 2.6	
26–35	5.82 ± 2.17	
35–45	6.25 ± 1.93	
>45	4.87 ± 2.36	
Marital status		0.017
Unmarried	4.99 ± 2.72	
Married	5.64 ± 2.25	
Educational level		0.810
School education or below	5.5 ± 2.5	
Bachelor degree or higher	5.44 ± 2.39	
Has a child		0.003
Yes	5.74 ± 2.2	
No	4.96 ± 2.69	
Underwent a cesarean		0.001
Yes	6.18 ± 2.12	
No	5.03 ± 2.47	
Total monthly family income		0.018
<1500	5.7 ± 1.98	
1500–3000	5.1 ± 2.42	
>3000	5.72 ± 2.51	
Would you choose spinal anesthesia over GA if you have CS in the future?		0.001
Yes	5.96 ± 2.29	
No	4.74 ± 2.41	
Hearing and source of Knowledge about Spinal anesthesia		0.001
No hearing about SA	4.03 ± 2.42	
Specialist (Gynecologist/Anesthesiologist)	6.55 ± 1.94	
Friend	5.16 ± 2.29	
Internet	6.07 ± 2.31	
Others	4.49 ± 2.31	

As shown in Table 4, several barriers were significantly associated with reluctance to opt for SA. Participants who did not plan to choose SA were more likely to believe it may cause permanent paralysis, involve painful injection, delay delivery, or harm the baby. Concerns about side effects such as chronic back pain and headaches were also more common in this group. In contrast, those intending to choose SA were more likely to disagree with these concerns. Differences in perceptions regarding hypotension were also statistically significant, although beliefs about nurse-administered SA and its timing during labor did not differ meaningfully between groups.

**Table 4:** Relationship between the plan to have spinal anesthesia and its perceived barriers (*n* = 415)

Barrier statement	Spinal anesthesia plan *N* (%)		*P* Value
	NO (*N* = 172)	YES (*N* = 243)	
1. Spinal anesthesia may cause permanent paralysis.			0.001
Agree	48(27.9)	33(13.6)	
Neutral	79(45.9)	100(41.2)	
Disagree	45(26.2)	110(45.3)	
2. Spinal anesthesia injection is very painful.			0.001
Agree	48(27.9)	31(12.8)	
Neutral	73(42.4)	96(39.5)	
Disagree	51(29.7)	116(47.7)	
3. Spinal anesthesia delays duration of delivery.			0.049
Agree	20(11.6)	15(6.2)	
Neutral	62(36)	76(31.3)	
Disagree	90(52.3)	152(62.6)	
4. Spinal anesthesia may harm the baby.			0.001
Agree	56(32.6)	12(4.9)	
Neutral	61(35.5)	66(27.2)	
Disagree	55(32)	165(67.9)	
5. Nurses can give spinal anesthesia.			0.086
Agree	26(15.1)	23(9.5)	
Neutral	60(34.9)	75(30.9)	
Disagree	86(50)	145(59.7)	
6. Spinal anesthesia should be given only before active labor.			0.356
Agree	89(51.7)	121(49.8)	
Neutral	51(29.7)	63(25.9)	
Disagree	32(18.6)	59(24.3)	
7. Spinal anesthesia may cause hypotension.			0.018
Agree	42(24.4)	58(23.9)	
Neutral	107(62.2)	126(51.9)	
Disagree	23(13.4)	59(24.3)	
8. Spinal anesthesia could cause chronic back pain after delivery.			0.001
Agree	94(54.7)	82(33.7)	
Neutral	56(32.6)	86(35.4)	
Disagree	22(12.8)	75(30.9)	
9. Spinal anesthesia may cause chronic headache.			0.001
Agree	61(35.5)	66(27.2)	
Neutral	80(46.5)	90(37)	
Disagree	31(18)	87(35.8)	
10. Spinal anesthesia does not completely relieve pain.			0.002
Agree	60(34.9)	54(22.2)	
Neutral	58(33.7)	75(30.9)	
Disagree	54(31.4)	114(46.9)	

## Discussion

This study aimed to evaluate women’s knowledge and awareness regarding spinal anesthesia for cesarean delivery, as well as to identify the underlying barriers that influence their decision-making about undergoing this procedure. knowledge about spinal anesthesia for cesarean delivery, revealing that most had a low to medium level of understanding. Specifically, 191 individuals (46.0%) demonstrated low knowledge, while 188 (45.3%) had a medium level, and only 36 participants (8.7%) exhibited a high level of knowledge. However, existing evidence indicates that greater efforts are required to enhance the level of knowledge among women. Consequently, healthcare professionals must address these knowledge gaps to promote a higher utilization rate of spinal anesthesia for cesarean sections.

The majority of participants in our study were aged 18–25 years (49.4%), followed by the 26–35 age group (29.2%). This age distribution reflects a young, reproductive-age population, consistent with demographic trends in Palestine [15]. Our findings indicate that married women, particularly those with children and a history of previous cesarean sections, tend to have higher levels of knowledge regarding spinal anesthesia. This observation is consistent with existing literature [9, 14, 16]. A study conducted by Endalew et al. [9]demonstrated that women with a prior history of surgical procedures exhibited a markedly higher level of understanding regarding anesthesia for cesarean sections. Henry and Nand [16] reported comparable results, highlighting that married woman with higher education levels, specifically those holding university degrees, as well as those with private insurance or receiving care at birth centers, possess a superior understanding of pain management during labor.

Family income was relatively balanced, with 15.4% low income, 42.7% moderate income, and 41.9% high income. Higher income groups tended to have higher knowledge scores. This finding consists with Makkah study, which found that women with higher family incomes had significantly better knowledge of SA [14]. Efforts to enhance awareness of spinal anesthesia should prioritize equitable dissemination of information across all socioeconomic strata, with particular attention to low-income populations who may encounter additional obstacles in accessing healthcare services [17]. 

This was very clear that participants who learned about SA from specialists’ doctors scored highest (6.55 ± 1.94), reinforcing the role of trusted healthcare providers in disseminating accurate information. This aligns with Ghanaian, where about two third of participated women cited doctors as their primary source of anesthesia knowledge [18]. 

Internet-based learning (6.07 ± 2.31) also emerged as a key contributor, mirroring trends in word, study conducting by Chin-Chung Tsai [19]. reviewing 46 studies from 1999 to 2009, focusing on learners’ self-efficacy in Internet-based learning environments. It highlights the positive role of self-efficacy in shaping attitudes and outcomes in such settings.

The results of our targeted evaluation of women’s knowledge of spinal anesthesia in relation to cesarean sections show that they have a good grasp of several important topics. These include the proper body posture during the injection, the patient’s level of consciousness during the process, the anesthesia’s effect, and the trained medical professional who is in charge of giving spinal anesthesia. Our results also point to significant gaps in the information regarding other important factors, such as injection site, the extent of anesthetic drug transmission to the newborn, and the suitability of spinal anesthesia for moms with coagulopathy or who are in labor. Notably, our results are consistent with a study conducted in Saudi Arabia, which reported similar findings. However, while 72.1% of the population in the Saudi study had accurate knowledge regarding the site of injection, our research showed a lower level of awareness, with 74.9% of respondents being ignorant of this information [14]. 

This study identifies numerous misconceptions and fears about spinal anesthesia (SA) during cesarean section that may influence its acceptance by women. Among the primary concerns that were brought up pertains to fear of complete paralysis with a significant proportion (43.1%) neutral on the matter, suggesting a lack of adequate knowledge, whereas 37.3% disagreed with this view. These findings are in accordance with previous studies demonstrating that misinformation regarding the long-term effects of SA makes parturients reluctant [1–3, 20, 21]. 

Pain during injection is another factor influencing the acceptability of SA because answers were more or less evenly distributed 40.7% were neutral in their opinion, whereas 40.2% denied that the injection is very painful. This observation is in agreement with findings noted in previous studies [14]. This suggests that while some women may perceive the procedure as uncomfortable, adequate pre-procedure counseling and pain management strategies could mitigate such concerns.

Unlike other studies, such as that among Saudi women [14], our study not only determined knowledge and perceived barriers but also examined their relationship to the type of anesthesia that women would prefer for future cesarean deliveries. We observed that greater knowledge and fewer misconceptions significantly corresponded to a desire for spinal anesthesia.

The fear that SA would delay delivery was, to a great extent, unfounded in this population, as 58.3% of those surveyed refuted this, confirming evidence that SA does not negatively affect labor progress. Similarly, the fear that SA leads to fetal harm was also, for the most part, debunked as 53% of those surveyed disagreed with this assertion.These findings contrast with reports from some regions where misconceptions about SA affecting neonatal outcomes contribute to lower utilization rates [14, 22]. 

Regarding the administration of SA, 55.7% of the participants disagreed that nursing should administer the procedure and wanted anesthesiologists or trained medical professionals to do so. This aligns with global standards advocating for trained anesthesia providers to ensure patient safety and procedural success [23]. 

An overwhelming majority (50.6%) believed that SA was to be administered only before active labor, reflecting potential lacunae in understanding its timing and effectiveness. Research has shown that these misconceptions can lead to unwarranted delays in the administration of anesthesia, which can enhance maternal pain and anxiety [24]. 

Physiologic concerns, like hypotension, were addressed mainly from a neutral perspective (56.1%), indicating doubt rather than outright rejection of risk incurred. Nonetheless, 42.4% of participants agreed that SA could cause chronic back pain, this misconception readily noted in the literature without accompanying supportive evidence. Another 30.6% were concerned with chronic headaches, which, though acknowledged as a potential complication, can often be managed effectively with proper post-procedural care [25–27]. 

Lastly, 46.9% of the respondents disagreed that SA does not totally relieve pain, attesting to the effectiveness of this technique for cesarean section. The 27.5% who believed that SA does not totally relieve pain, however, indicate the need for adequate patient education regarding its analgesic effect.

These findings emphasize the significant role that can be played by healthcare professionals in bridging knowledge gaps and dispelling misconceptions regarding SA. Proper counseling and preoperative counseling are essential in alleviating unfounded fears and ensuring informed decision-making among pregnant women. Barriers need to be removed and awareness increased in order to optimize the uptake of SA for cesarean deliveries, thereby improving maternal experience and surgical outcomes.

This study has a number of limitations. Firstly, the cross-sectional design does not allow for the determination of causal links between levels of knowledge and preferences for anesthesia. Secondly, the utilization of self-reported information potentially brings about recall or social desirability bias, especially when it comes to the participants’ previous exposure to anesthesia and the expressed attitudes. Thirdly, although the research involved a rather large sample, regional differences between various governorates in Palestine were not thoroughly investigated, which can compromise the external validity of the findings.

## Conclusions 

This study reveals a low to moderate overall level of knowledge among women regarding spinal anesthesia (SA) for cesarean delivery, with significant misconceptions and fears guiding their choices. Key demographic and clinical vaiables such as age, marital status, number of children, number of cesarean sections, income level, and source of information were significantly associated with knowledge levels. Women who had prior cesarean deliveries, higher income, or received information from specialists demonstrated notably better understanding.

Misconceptions and fears, such as concerns about paralysis, pain related to the injection, or long-term back pain, showed a strong correlation with reluctance to accept spinal anesthesia. womens with poorer knowledge levels were more likely to confirm these fears, highlighting the impact of misinformation on decision making. It is essential to adopt targeted educational interventions that specifically address these misconceptions, provided by trained healthcare professionals, to improve the acceptability of spinal anesthesia in women undergoing cesarean delivery.

## Data Availability

The dataset used in this study will be available from corresponding author upon a reasonable request.

## Abbreviations

CS: Cesarean section
GA: General anesthesia
RA: Regional anesthesia
SA: Spinal anesthesia
OECD: Organization for economic co-operation and development
SPSS: Statistical package for the social sciences
